# Therapeutic Effects of Glutamic Acid in Piglets Challenged with Deoxynivalenol

**DOI:** 10.1371/journal.pone.0100591

**Published:** 2014-07-01

**Authors:** Miaomiao Wu, Hao Xiao, Wenkai Ren, Jie Yin, Bie Tan, Gang Liu, Lili Li, Charles Martin Nyachoti, Xia Xiong, Guoyao Wu

**Affiliations:** 1 Scientific Observing and Experimental Station of Animal Nutrition and Feed Science in South-Central China, Ministry of Agriculture, Hunan Provincial Engineering Research Center of Healthy Livestock Key Laboratory of Agro-Ecological Processes in Subtropical Region, Institute of Subtropical Agriculture, Chinese Academy of Scienses, Changsha, Hunan, China; 2 University of Chinese Academy of Sciences, Beijing, China; 3 Department of Animal Science, University of Manitoba, Winnipeg, Canada; 4 Department of Animal Science, Texas A&M University, College Station, Texas, United State of America; Wageningen UR Livestock Research, Netherlands

## Abstract

The mycotoxin deoxynivalenol (DON), one of the most common food contaminants, primarily targets the gastrointestinal tract to affect animal and human health. This study was conducted to examine the protective function of glutamic acid on intestinal injury and oxidative stress caused by DON in piglets. Twenty-eight piglets were assigned randomly into 4 dietary treatments (7 pigs/treatment): 1) uncontaminated control diet (NC), 2) NC+DON at 4 mg/kg (DON), 3) NC+2% glutamic acid (GLU), and 4) NC+2% glutamic acid + DON at 4 mg/kg (DG). At day 15, 30 and 37, blood samples were collected to determine serum concentrations of CAT (catalase), T-AOC (total antioxidant capacity), H_2_O_2_ (hydrogen peroxide), NO (nitric oxide), MDA (maleic dialdehyde), DAO (diamine oxidase) and D-lactate. Intestinal morphology, and the activation of Akt/mTOR/4EBP1 signal pathway, as well as the concentrations of H_2_O_2_, MDA, and DAO in kidney, liver and small intestine, were analyzed at day 37. Results showed that DON significantly (P<0.05) induced oxidative stress in piglets, while this stress was remarkably reduced with glutamic acid supplementation according to the change of oxidative parameters in blood and tissues. Meanwhile, DON caused obvious intestinal injury from microscopic observations and permeability indicators, which was alleviated by glutamic acid supplementation. Moreover, the inhibition of DON on Akt/mTOR/4EBP1 signal pathway was reduced by glutamic acid supplementation. Collectively, these data suggest that glutamic acid may be a useful nutritional regulator for DON-induced damage manifested as oxidative stress, intestinal injury and signaling inhibition.

## Introduction

Deoxynivalenol (DON) is the most pervasive mycotoxin, which are found worldwide in various foods and animal feeds [1]–[3]. The initial adverse effects observed after DON exposure are reduced feed intake, emesis, diarrhea, and anorexia [4]. DON becomes a serious problem in animal production worldwide, especially in pigs, because of its adverse effects on brain, liver, kidney, and mostly gastrointestinal tract [5]–[10]. These adverse effects include the inhibitions of DNA, RNA and protein synthesis, and lesions in the gastrointestinal tract, affecting its barrier function, as well as modulation of the anti-oxidative system [9], [11]–[14]. Although DON causes a big economical loss to swine production, little has been done to investigate the nutritional strategy that may be useful in protecting pigs from the damage caused by consuming DON in contaminated diets.

Glutamic acid, a functional amino acid, plays various crucial roles in the intestinal tract, including (1) substrate for various metabolic pathways [15], [16], (2) energy source for intestinal mucosa [17], (3) mediator for cell signaling [18], [19], (4) regulator for oxidative reactions [20], [21], as well as immune responses and barrier function [22], [23].

Considering the known functions of glutamic acid in intestine, we hypothesized that dietary glutamic acid supplementation may ameliorate the toxic effects of DON. Therefore, the objective of the current study was to investigate the effects of glutamic acid supplementation on the oxidative stress, intestinal barrier loss and protein inhibition induced by DON in piglets.

## Materials and Methods

### Preparation of DON-contaminated feed

*Fusarium graminearum*R6576, producing DON, was kindly provided by Huazhong Agricultural University (Wuhan, China). The DON-contaminated feed was prepared according to the previous reports from our group [13], [14].

### Experimental design

This study was conducted according to the guidelines of the Declaration of Helsinki, and animal protocols were approved by the animal welfare committee of the Institute of Subtropical Agriculture, Chinese Academy of Sciences. A total of 28 pigs (Landrace×Large White) (ZhengHong Co., China) weaned at 28 days of age with a mean body weight (BW) of 12.3±2.3 kg, were subjected to four treatments (n = 7 per group): 1) NC group, weaning pigs received uncontaminated basal diets; 2) DON group, weaning pigs received 4 mg/kg deoxynivalenol-contaminated diets; 3) GLU group, weaning pigs received dietary 2% glutamic acid supplementation; 4) DG group, weaning pigs received 4 mg/kg deoxynivalenol diets and dietary 2% glutamic aid supplementation. The concentrations of DON in different groups shown in Table 1. The basal diets [13] were prepared from corn, soybean meal, wheat bran, limestone, CaHPO_4_, salt, and additive premix to meet or exceed the nutritional requirements of growing pig as recommended by the NRC (1998) (Table 2). Glutamic acid was added to the feed and mixed uniformly.

**Table 1:** Deoxynivalenol content in different dietary groups.

Item	NC group	DON group	GLU group	DG group
Content(mg/kg)	1.09±0.04	3.97±0.05	1.01±0.01	4.04±0.03

Pigs (7 pigs/group) were fed with uncontaminated basal diet (NC), or basal diet contaminated with deoxynivalenol at dose of 4 mg/kg (DON), or uncontaminated basal diet supplemented with 2% glutamic acid (GLU), or DON diet supplemented with 2% glutamic acid (DG). Deoxynivalenol content was determined using an assay kit in accordance with the manufacturer’s instructions (Cusabio, China).

**Table 2:** Composition and nutrient levels of basal diet (as-fed basis)^1^.

Ingredients	Contents(%)	Nutrition levels	Contents(%)
Corn	61.25	CP	19.00
Soybean meal	15.79	DE/(MJ/Kg)	14.11
Extruded-soybean	10.00	Calcium	0.80
Imported fish meal	5.00	TP	0.63
Wheat bran	3.00	AP	0.40
Soybean oil	1.74	Lysine	1.15
Premix^2^	1.00	Methionie+cysteine	0.67
Limestone	0.98	Threonine	0.77
CaHPO_4_	0.78	Tryptophan	0.22
Salt	0.37		
Lysine-HCl	0.09		
Total	100.00		

1 NC = uncontaminated basal diet, DON = basal diet contaminated with deoxynivalenol (4 mg/kg), GLU = uncontaminated basal diet supplemented with 2% glutamic acid; DG = DON diet supplemented with 2% glutamic acid.

2 Providing the following amount of vitamins and minerals per kilogram on an as-fed basis: Zn (ZnO), 50 mg; Cu (CuSO_4_), 20 mg; Mn (MnO), 55 mg; Fe (FeSO_4_), 100 mg; I (KI), 1 mg; Co (CoSO_4_), 2 mg; Se (Na_2_SeO_3_), 0.3 mg; vitamin A, 8,255 IU; vitamin D_3_, 2,000 IU; vitamin E, 40 IU; vitamin B_1_, 2 mg; vitamin B_2_, 4 mg; pantothenic acid, 15 mg; vitamin B_6_, 10 mg; vitamin B_12_, 0.05 mg; vitamin PP, 30 mg; folic acid, 2 mg; vitamin K_3_, 1.5 mg; biotin, 0.2 mg; choline chloride, 800 mg; and vitamin C, 100 mg. The premix did not contain additional copper, zinc, antibiotics, or probiotics.

CP: Crude Protein; DE: Digestible Energy; TP: Total Phosphorus; AP: Available Phosphorus.

The experiment was arranged as a randomized design. Experiment animals were allowed free access to water throughout the experimental period. The trial period lasted for 37 days. At day 37, piglets were anesthetized with sodium pentobarbital and killed by jugular puncture. Jejunum and ileum were collected for intestinal mucosal morphology, diamine oxidase (DAO), maleic dialdehyde (MDA), total antioxidant capacity (T-AOC), and some signal proteins analysis. Liver and kidney also were collected to detect MDA, DAO, hydrogen peroxide (H_2_O_2_) and T-AOC. Serum were prepared on day 15, 30 and 37 for catalase (CAT), MDA, nitric oxide (NO), H_2_O_2_ and amino acids levels determination. Plasma were prepared on day 15, 30 for D-lactate and DAO determination.

### Antioxidation capacity, D-lactate and amino acids levels analysis

Spectrophotometric kits was used to analyze several parameters in samples, including CAT activities, and MDA, NO and H_2_O_2_ levels in the serum; T-AOC activity; and MDA and H_2_O_2_ levels in the liver and kidney; as well as T-AOC activity and MDA level in the jejunum and ileum. The protocol was conducted according to the manufacturer’s instructions (Nanjing Jiancheng Biotechnology Institute, China). The reaction system for determining DAO concentration included 0.1 ml (4 µg) horseradish peroxidase solution (Sigma), 3 ml 0.2 M, pH 7.2 PBS, 0.1 ml O-dianisidine methanol solution (500 µg of O-dianisidine) (Sigma), 0.5 ml sample, and 0.1 ml substrate solution (175 µg of cadaverine dihydrochloride) (Sigma). Then the processed samples were incubated in an incubator chamber at 37°C for 30 min and measured at 436 nm by UV/visible spectrophotometer-UV–2450 (SHIMADZU, Japan) [24]. Plasma D-lactate was determined using assay kit in accordance with the manufacturer’s instruction (BioVision Inc., America). The serum was separated with centrifugation at 3,500×g for 15 min at 4°C and stored at −20°C. Concentrations of amino acids in the serum were detected via LC-MS/MS (HPLC Ultimate3000 and 3200 Q TRAP LC-MS/MS). Serum amino acids were analyzed with isotope dilution liquid chromatography-mass spectrometry methods by Beijing Amino Medical Research CO., LTD, Beijing, China.

### Histomorphometry determination

Jejunum and ileum morphology were analyzed using hemotoxylin eosin (HE) stain morphology. After dehydration, embedding, sectioning and staining, villous height, crypt depth, goblet cell and lymphocyte counts were measured with computer-assisted microscopy (Micrometrics TM; Nikon ECLIPSE E200, Japan). For each intestinal segment, nine villus heights and crypt depths were measured using Motic Images Advanced 3.2. Measurements were taken in ten well-oriented villi and ten crpyts from each intestinal tissue section of each piglet.

### Western blot analysis

Western blot analysis was conducted according to a previous study [25]. Briefly, the equal amounts of proteins obtained from the ileum and jejunum samples were separated by a reducing SDS-PAGE electrophoresis. The proteins were transferred onto PVDF membranes (Millipore, MA, USA) and blocked with 5% non-fat milk in Tris-Tween buffered saline buffer (20 mM Tris, pH 7.5,150 mM NaCl, and 0.1% Tween-20) for 3 hr. The following primary antibodies were incubated overnight at 4°C with gentle rocking: β-actin (Santa Vruz, 1∶400), total 4EBP1 (T-4EBP1, Cell Signaling, 1∶1000), phosphorylated 4EBP1 (Ser 65) (Cell Signaling, 1∶1000), total Akt (T-Akt, Cell Signaling, 1∶1000), phosphorylated Akt (Ser 473) (Cell Signaling, 1∶1000), total mTOR (T-mTOR, Cell Signaling, 1∶1000) or phosphorylated mTOR (Ser 2448) (Cell Signaling, 1∶1000). Then, the HRP-conjugated secondary antibodies were subsequently incubated for 1 hr at room temperature before developing the blots using Alpha Imager 2200 software (Alpha Innotech Corporation, CA, USA). We digitally quantified the resultant signals and normalized the data to the actin abundance. β-actin was used as an internal loading control for cytoplasmic protein fractions.

### Statistical Analysis

Data are expressed as the mean ± standard error of the mean (SEM). All statistical analyses were performed using the SPSS17.0 software (Chicago, IL, USA). Group comparisons were performed using Tukey multiple range test. Differences were considered significant at P≤0.05.

## Results

### Anti-oxidative capacity

**Figure 1:**
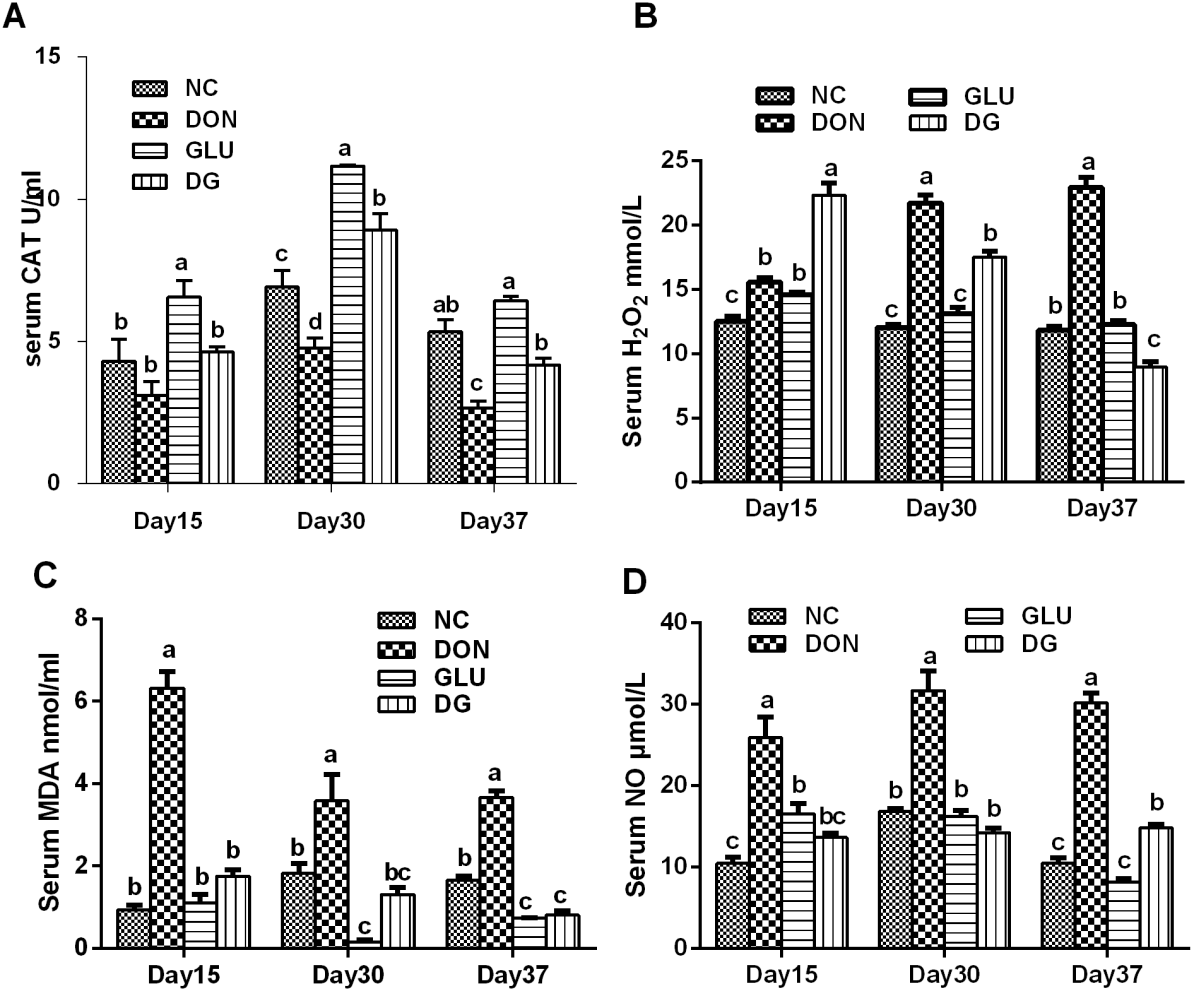
Blood anti-oxidative capacity in each group. A: CAT activity in each group. B: H_2_O_2_ concentration in each group. C: MDA concentration in each group. D: NO concentration in each group. Dietary treatments were NC, an uncontaminated basal diet, DON, the basal contaminated with 4 mg/kg deoxynivalenol, GLU, uncontaminated basal diet with 2% (g/g) glutamic acid supplementation, and DG, deoxynivalenol-contaminated (4 mg/kg) basal diet with 2% (g/g) glutamic acid supplementation. Data are presented as means ± SEM, n = 7, with a–c used to indicate a statistically significant difference (P<0.05, one way ANOVA method). CAT: catalase (U/ml); H_2_O_2_: hydrogen peroxide (mmol/L); MDA: methane dicarboxylic aldehyde (nmol/ml); NO: nitric oxide (µmol/L).

As shown in Figure 1A, DON significantly (P<0.05) decreased serum CAT activity compared with controls at day 30 and 37. This decrease was reversed by glutamic acid supplementation at day 30 and 37(P<0.05) (Figure 1A). The effect of glutamic acid on CAT is obvious because a higher CAT activity in the serum was observed at day 15(P<0.05), 30(P<0.05) and 37(P>0.05), than the normal group (Figure 1A).

Serum H_2_O_2_ levels in DON were higher (P<0.05) than those in the NC group at day 15, 30 and 37(Figure 1B). Glutamic acid supplementation significantly reduced (P<0.05) the serum H_2_O_2_ concentration at day 30 and 37 (Figure 1B). Interestingly, a higher level of H_2_O_2_ was observed in the piglets fed the DG at day 15 (Figure 1B).

Serum MDA (Figure 1C) and NO (Figure 1D) concentrations were higher (P<0.05) in piglets fed the DON diet than those fed the NC diet. These increases were remarkably (P<0.05) alleviated by glutamic acid supplementation.

**Figure 2:**
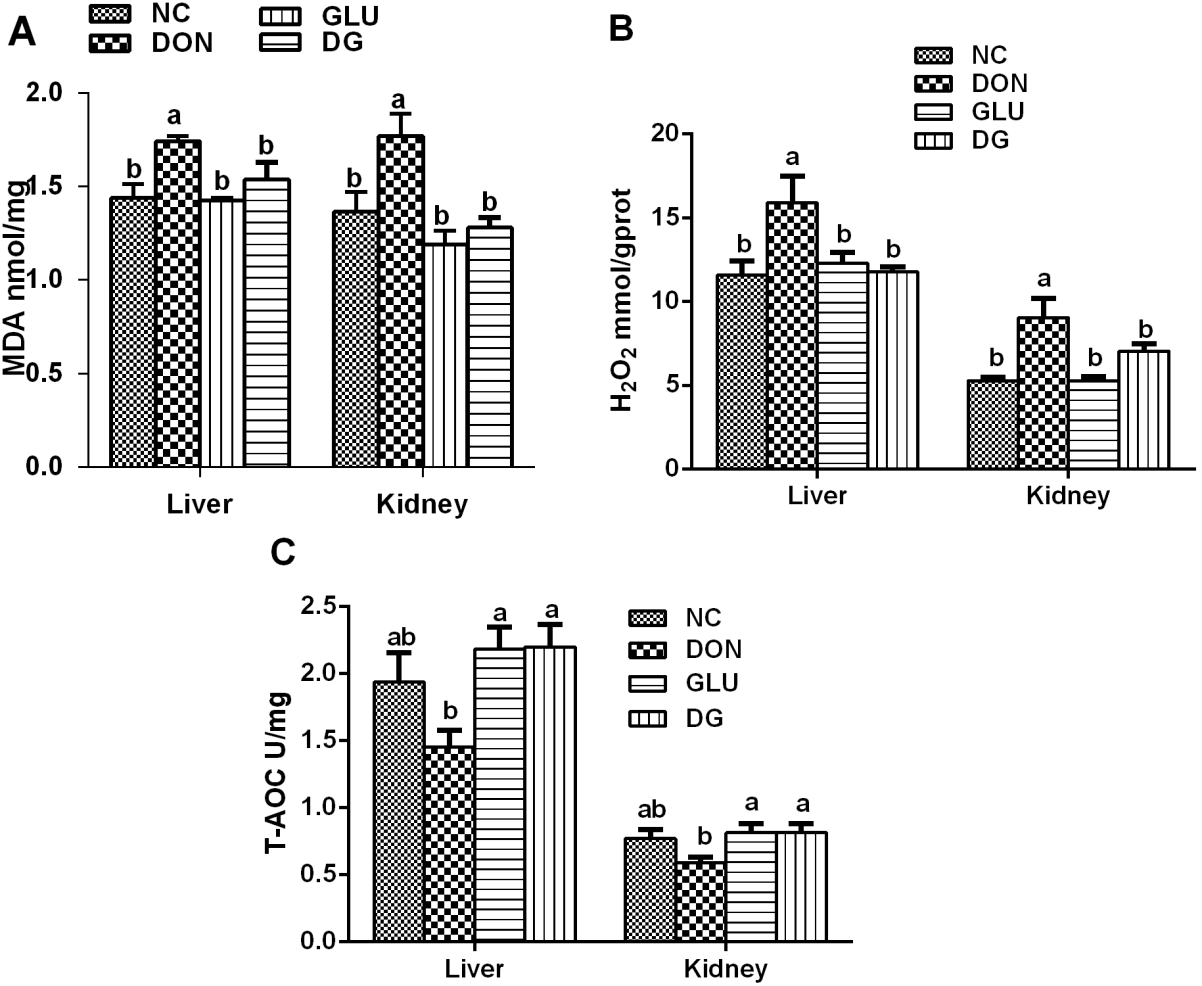
Liver and kidney anti-oxidative capacity in each group. A: MDA concentrations of liver and kidney in each group. B: H_2_O_2_ concentrations of liver and kidney in each group. C: T-AOC of liver and kidney in each group. Dietary treatments were NC, an uncontaminated basal diet, DON, the basal contaminated with 4 mg/kg deoxynivalenol, GLU, uncontaminated basal diet with 2% (g/g) glutamic acid supplementation, and DG, deoxynivalenol-contaminated (4 mg/kg) basal diet with 2% (g/g) glutamic acid supplementation. Data are presented as means ± SEM, n = 7, with a–b used to indicate a statistically significant difference (P<0.05, one way ANOVA method). MDA: methane dicarboxylic aldehyde (nmol/ml); H_2_O_2_: hydrogen peroxide (mmol/L); T-AOC: total antioxidant capacity (U/mg).

Piglets consuming the DON diet had significantly (P<0.05) increased production of MDA (Figure 2A) and H_2_O_2_ (Figure 2B) in the liver and kidney; while had little effect on the T-AOC activity in the liver and kidney, compared to those fed the NC diet. The production of MDA (Figure 2A) and H_2_O_2_ (Figure 2B) in the liver and kidney was significantly lowered with glutamic acid supplementation. Meanwhile, in the liver, the T-AOC activity in DG group was higher (P<0.05) than those in the DON group (Figure 2C).

**Figure 3:**
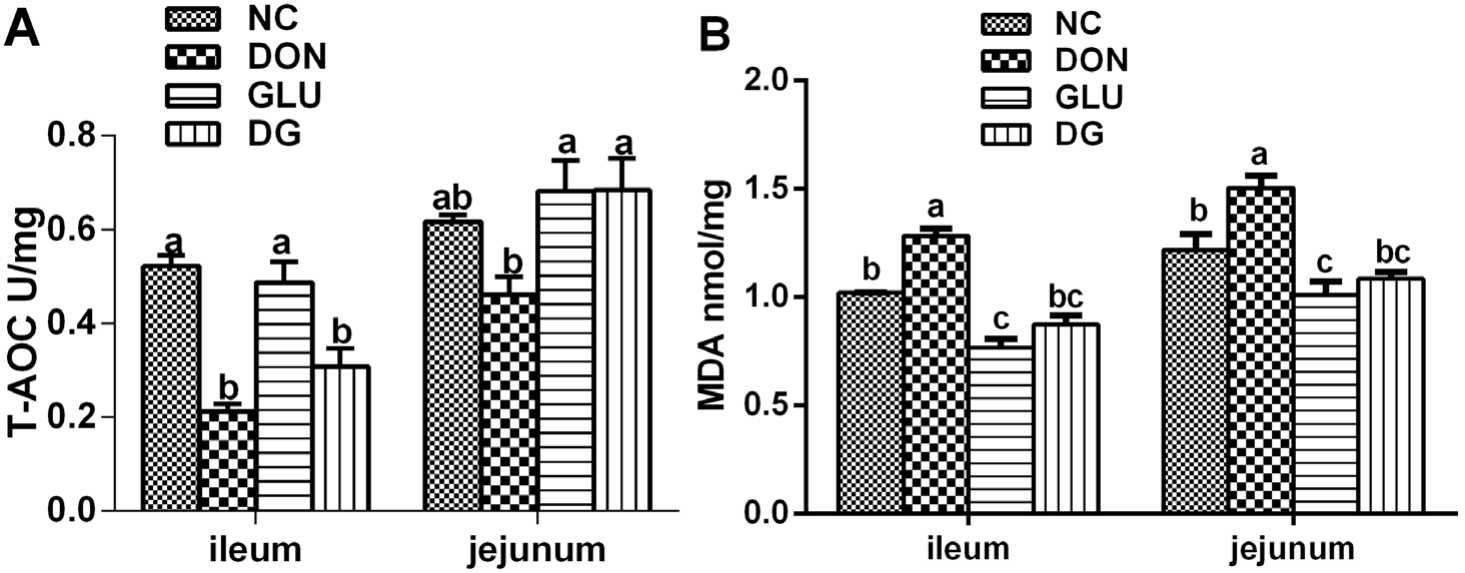
Ileum and jejunum anti-oxidative capacity in each group. A: T-AOC of ileum and jejunum in each group. B: MDA concentrations of ileum and jejunum in each group. Dietary treatments were NC, an uncontaminated basal diet, DON, the basal contaminated with 4 mg/kg deoxynivalenol, GLU, uncontaminated basal diet with 2% (g/g) glutamic acid supplementation, and DG, deoxynivalenol-contaminated (4 mg/kg) basal diet with 2% glutamic acid supplementation. Data are presented as means ± SEM, n = 7, with a–c used to indicate a statistically significant difference (P<0.05, one way ANOVA method). MDA: methane dicarboxylic aldehyde (nmol/ml); T-AOC: total antioxidant capacity (U/mg).

In the ileum, the T-AOC concentration was lower (P<0.05) for piglets fed the DON-contaminated diets than those fed the NC diets, while DON had little effect on the jejunum (Figure 3A). In the ileum and jejunum, MDA content was higher (P<0.05) in the piglets fed the DON diet than in those fed the other diets (Figure 3B). The beneficial function of glutamic acid supplementation was observed again by increased T-AOC activity in DG group in the jejunum, and decreased MDA levels in DG group in the ileum and jejunum, compared to the DON groups. (P<0.05) (Figure 3).

### DAO activity, D-lactate levels and Histological Evaluation

**Figure 4:**
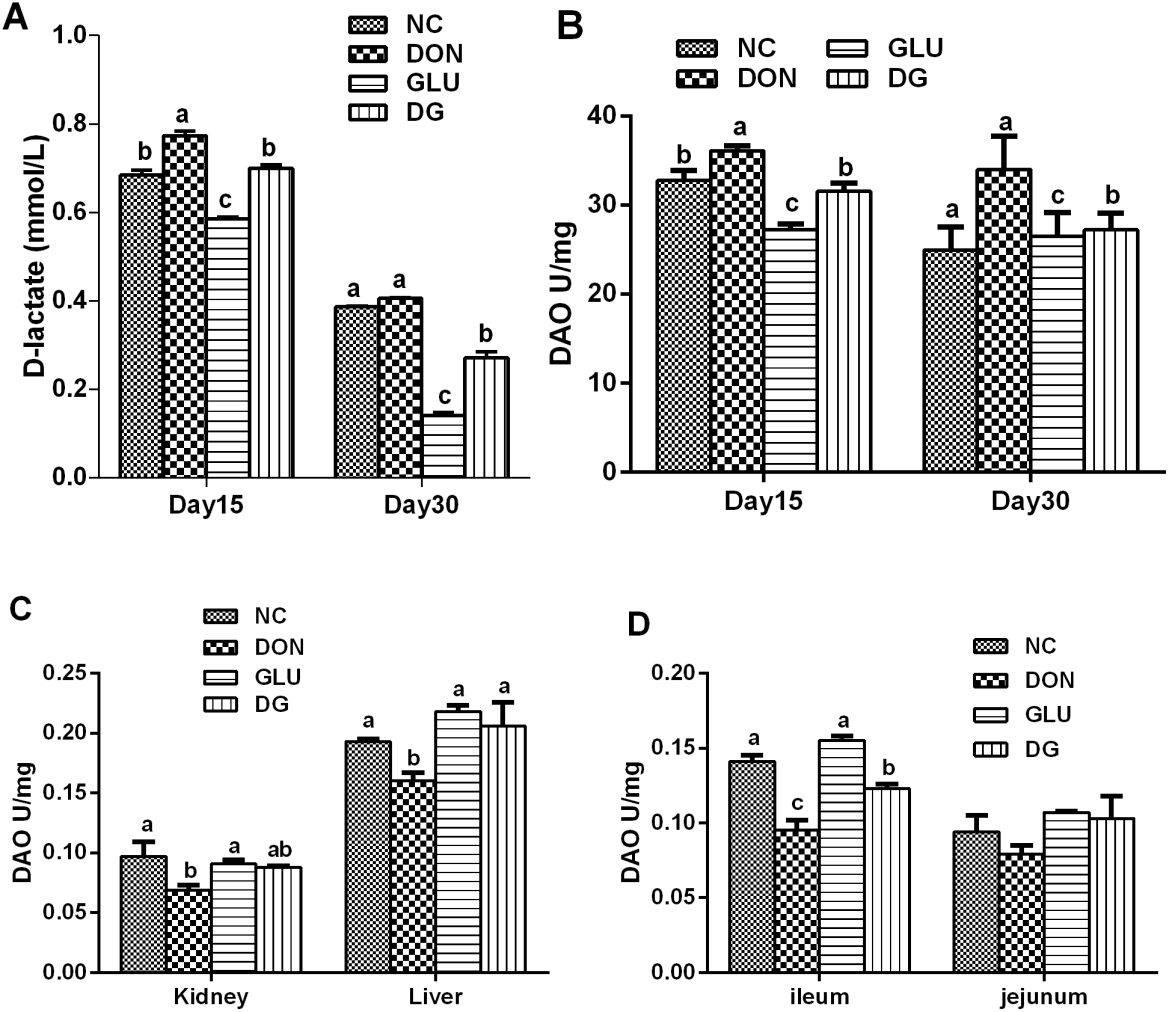
Plasma DAO activity and D-lactate levels in each group. A: Plasma D-lactate levels in each group on day 15 and day 30. B: Plasma DAO activity in each group on day 15 and day 30. C: DAO activity of kidney and liver in each group. D: DAO activity of ileum and jejunum in each group. Dietary treatments were NC, an uncontaminated basal diet, DON, the basal contaminated with 4 mg/kg deoxynivalenol, GLU, uncontaminated basal diet with 2% (g/g) glutamic acid supplementation, and DG, deoxynivalenol-contaminated (4 mg/kg) basal diet with 2% (g/g) glutamic acid supplementation. Data are presented as means ± SEM, n = 7, with a–d used to indicate a statistically significant difference (P<0.05, one way ANOVA method). DAO: dianine oxidase (U/mg).

DAO activity in serum and tissue, and D-lactate levels in serum are useful biomarkers for evaluating the integrity of the gastrointestinal tract [26]. As shown in Figure 4, feeding the DON diet increased the plasma D-lactate concentration by 13.0% at day 15, while DON had little effect on plasma D-lactate concentration at day 30, compared with the piglets feed the NC diet. However, supplementing the DON-contaminated diet with 2% glutamic acid reduced (P<0.05) plasma D-lactate level by 9.4% and 33.0% at day 15 and 30, respectively. DON significantly increased the plasma DAO activity by 10.2% (day 15), and 36.1% (day 30), while DON reduced (P<0.05) the DAO activity in the liver, kidney, and ileum by 17.1%, 28.9%, and by 32.6% on day 37, respectively, compared with the control. However, supplementing the DON-contaminated diet with 2% glutamic acid reduced (P<0.05) plasma DAO activity by 12.7% and 20.0%, at day 15 and 30, respectively, while increased DAO activity by 28.8%, 27.5%, and 29.5% 8 in the liver, kidney, and ileum, at day 37, respectively.

**Figure 5:**
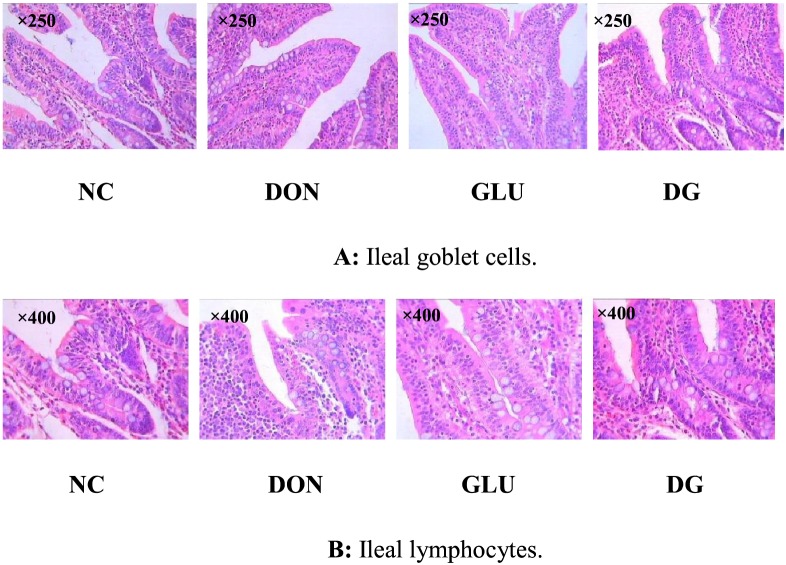
Image of ileal goblet cells and ileal lymphocytes. A: Ileal goblet cells (×250). B: Ileam lymphocytes (×400). Dietary treatments were NC, an uncontaminated basal diet, DON, the basal contaminated with 4 mg/kg deoxynivalenol, GLU, uncontaminated basal diet with 2% (g/g) glutamic acid supplementation, and DG, deoxynivalenol-contaminated (4 mg/kg) basal diet with 2% (g/g) glutamic acid supplementation. n = 7 for treatments.

**Figure 6:**
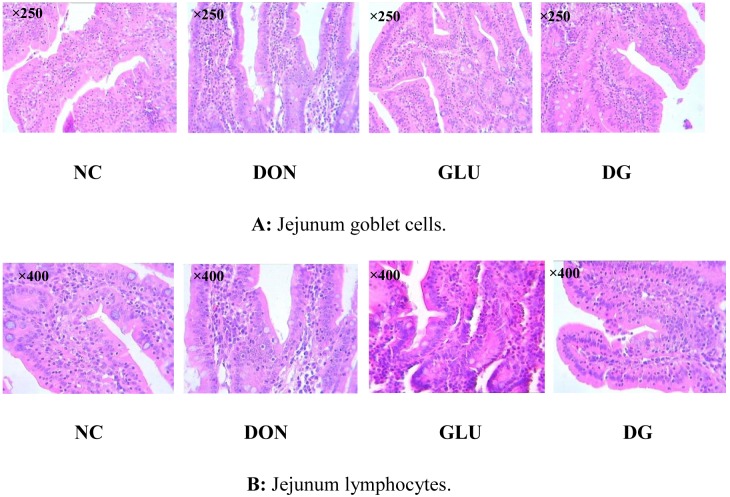
Image of jejunum goblet cells and jejunum lymphocytes. A: Jejunum goblet cells (×250). B: Jejunum lymphocytes (×400). Dietary treatments were NC, an uncontaminated basal diet, DON, the basal contaminated with 4 mg/kg deoxynivalenol, GLU, uncontaminated basal diet with 2% (g/g) glutamic acid supplementation, and DG, deoxynivalenol-contaminated (4 mg/kg) basal diet with 2% (g/g) glutamic acid supplementation. n = 7 for treatments.

As shown in Figures 5 and 6, Tables 3, there was no histological damage in the piglets fed the NC diet. However, compared with the NC diet, feeding the DON diet reduced (P<0.05) villus height in the ileum and jejunum, and increased lymphocytes (P<0.05) in the ileum and jejunum, and goblet cells (P<0.05) in the jejunum. Feeding the DG diet increased (P<0.05) villus height in the ileum and jejunum, and reduced (P<0.05) lymphocytes, goblet cells, and crypt depth in the jejunum.

**Table 3:** Effect of glutamic acid supplementation on ileal and jejunum morphology in piglets fed a deoxynivalenol-contaminated diet.

Variable		Diets^1^			
		NC	DON	GLU	DG
Goblet cell	ileal	22.3±3.7	17.3±4.6	16.5±0.7	18.8±1.4
	jejunum	10.0±2.5^b^	17.7±4.1^a^	9.25±1.55^b^	11.0±1.1^b^
villus height(VH, µm)	ileal	263±8^a^	171±11^c^	271±8^a^	202±4^b^
	jejunum	245±7^ab^	200±11^b^	252±12^ab^	234±16^a^
crypt depth(CD, µm)	ileal	118±14	111±4	114±7	99.6±1.9
	jejunum	116±2^ab^	156±19^a^	106±6^b^	110±8^b^
lymphocyte	ileal	181±2^c^	232±6^a^	171±3^c^	214±2^b^
	jejunum	195±19^c^	256±6^a^	203±16^c^	218±18^b^
villus height/crypt depth(VH/CD)	ileal	2.32±0.15^a^	1.73±0.07^b^	2.40±0.09^a^	1.95±0.01^ab^
	jejunum	2.11±0.10^ab^	1.86±0.11^b^	2.25±0.06^a^	2.13±0.03^ab^

n = 7. ^1^NC = uncontaminated basal diet, DON = basal diet contaminated with deoxynivalenol (4 mg/kg), GLU = uncontaminated basal diet supplemented with 2% glutamic acid; DG = DON diet supplemented with 2% glutamic acid. Data are presented as means ± SEM, n = 7, with a–c used to indicate a statistically significant difference (P<0.05, one way ANOVA method).

### Intestinal signaling and amino acid profile

Feeding the DON diet generally reduced amino acid contents in the small intestine (Tables 4, 5, and 6), and these effects were significant (P<0.05) for L-isoleucine, L-methionine, L-threonine, L-asparagine, L-glutamic acid, and L-proline. However, feeding the DG diet increased the intestinal contents of these amino acids (P<0.05).

**Table 4:** Effect of dietary supplementation with glutmic acid on serum amino acids levels (µg/ml) in piglets fed a deoxynivalenol-contaminated diet on day 15.

Item	NC	DON	GLU	DG
L-histidine	18.99±1.62^ab^	13.47±0.46^c^	22.91±1.08^a^	16.55±1.51^bc^
L-isoleucine	13.10±1.11	13.73±1.04	14.02±0.58	11.85±0.87
L-leucine	25.85±2.03^a^	19.64±1.17^b^	23.32±0.74^ab^	22.53±0.45^ab^
L-methionine	5.05±0.59^a^	0.41±0.05^c^	4.46±0.19^ab^	3.21±0.14^b^
L-threonine	6.75±0.69^ab^	4.68±0.46^b^	8.84±0.76^a^	6.96±0.55^ab^
L-asparagine	7.75±0.66^a^	5.40±0.40^b^	7.80±0.31^a^	6.52±0.49^ab^
L-aspartic acid	0.49±0.03	0.42±0.02	0.46±0.03	0.42±0.03
L-glutamic acid	9.28±0.52^a^	7.74±0.11^b^	9.26±0.67^a^	8.99±0.42^ab^
L-ornithine	18.48±3.17^a^	13.01±1.53^ab^	14.06±1.32^ab^	12.05±0.68^b^
L-cystine	1.50±0.27^a^	1.10±0.15^ab^	0.83±0.22^ab^	0.57±0.25^b^
L-carnosine	0.45±0.04^a^	0.30±0.04^b^	0.26±0.03^b^	0.24±0.03^b^
L-proline	46.02±3.85^a^	39.36±2.77^ab^	32.66±2.02^b^	37.73±1.56^ab^

NC = uncontaminated basal diet, DON = basal diet contaminated with deoxynivalenol (4 mg/kg), GLU = uncontaminated basal diet supplemented with 2% glutamic acid.; DG = DON diet supplemented with 2% glutamic acid. Data are presented as means ± SEM, n = 7, with a–b used to indicate a statistically significant difference (P<0.05, one way ANOVA method).

**Table 5:** Effect of dietary supplementation with glutmic acid on serum amino acids levels (µg/ml) in piglets fed a deoxynivalenol-contaminated diet on day 30.

Item	NC	DON	GLU	DG
L-histidine	29.41±1.57	23.02±1.06	22.19±1.59	22.04±1.03
L-isoleucine	17.22±1.16^a^	12.64±0.73^b^	16.63±0.86^a^	14.33±0.42^ab^
L-leucine	30.64±3.05^a^	23.61±1.29^b^	30.18±0.91^a^	26.41±1.17^ab^
L-methionine	0.72±0.07	0.51±0.03	0.84±0.09	0.70±0.06
L-threonine	12.99±0.71	10.50±0.97	11.04±0.96	11.79±0.70
L-asparagine	10.20±1.37	9.05±0.93	9.70±0.42	8.45±0.47
L-aspartic acid	0.71±0.05	0.61±0.03	0.64±0.02	0.66±0.07
L-glutamic acid	10.82±0.88^a^	8.00±0.34^b^	10.84±0.27^a^	9.63±0.54^ab^
L-Ornithine	21.37±4.23^ab^	13.01±1.06^b^	28.23±1.85^a^	23.98±2.70^a^
L-cystine	1.54±0.35^a^	0.39±0.03^b^	1.39±0.48^a^	0.44±0.05^b^
L-carnosine	0.53±0.05	0.40±0.03	0.40±0.04	0.40±0.02
L-proline	35.61±3.18	36.39±0.50	45.51±1.56	36.28±0.63

NC = uncontaminated basal diet, DON = basal diet contaminated with deoxynivalenol (4 mg/kg), GLU = uncontaminated basal diet supplemented with 2% glutamic acid; DG = DON diet supplemented with 2% glutamic acid. Data are presented as means ± SEM, n = 7, with a–b used to indicate a statistically significant difference (P<0.05, one way ANOVA method).

**Table 6:** Effect of dietary supplementation with glutmic acid on serum amino acids levels in piglets fed deoxynivalenol-contaminated feed on day 37.

Item	NC	DON	GLU	DG
L-histidine	34.99±1.68^a^	23.27±2.65^b^	31.10±2.91^ab^	34.62±1.63^a^
L-isoleucine	11.96±0.96	8.91±0.86	12.32±0.18	10.83±0.32
L-leucine	27.59±2.01	26.60±2.22	28.03±1.08	24.28±1.69
L-methionine	0.36±0.01^bc^	0.26±0.02^c^	0.56±0.04^a^	0.38±0.02^b^
L-threonine	9.54±0.54^ab^	6.48±0.63^c^	11.18±0.49^a^	7.96±0.46^bc^
L-asparagine	8.33±0.56^a^	4.70±0.26^b^	8.13±0.57^a^	8.05±0.53^a^
L-aspartic acid	0.58±0.06	0.54±0.07	0.54±0.05	0.47±0.04
L-glutamic acid	10.24±0.51^a^	3.45±0.29^c^	9.29±0.19^ab^	7.68±0.52^b^
L-Ornithine	25.77±3.43	18.93±1.81	23.63±4.35	17.11±1.22
L-cystine	1.07±0.26	1.27±0.24	0.96±0.17	0.92±0.09
L-carnosine	0.45±0.03^a^	0.32±0.04^b^	0.63±0.06^ab^	0.52±0.06^ab^
L-proline	35.10±2.01^a^	25.89±0.92^c^	31.29±1.06^ab^	30.16±1.00^b^

NC = uncontaminated basal diet, DON = basal diet contaminated with deoxynivalenol (4 mg/kg), GLU = uncontaminated basal diet supplemented with 2% glutamic acid; DG = DON diet supplemented with 2% glutamic acid. Data are presented as means ± SEM, n = 7, with a–c used to indicate a statistically significant difference (P<0.05, one way ANOVA method).

**Figure 7:**
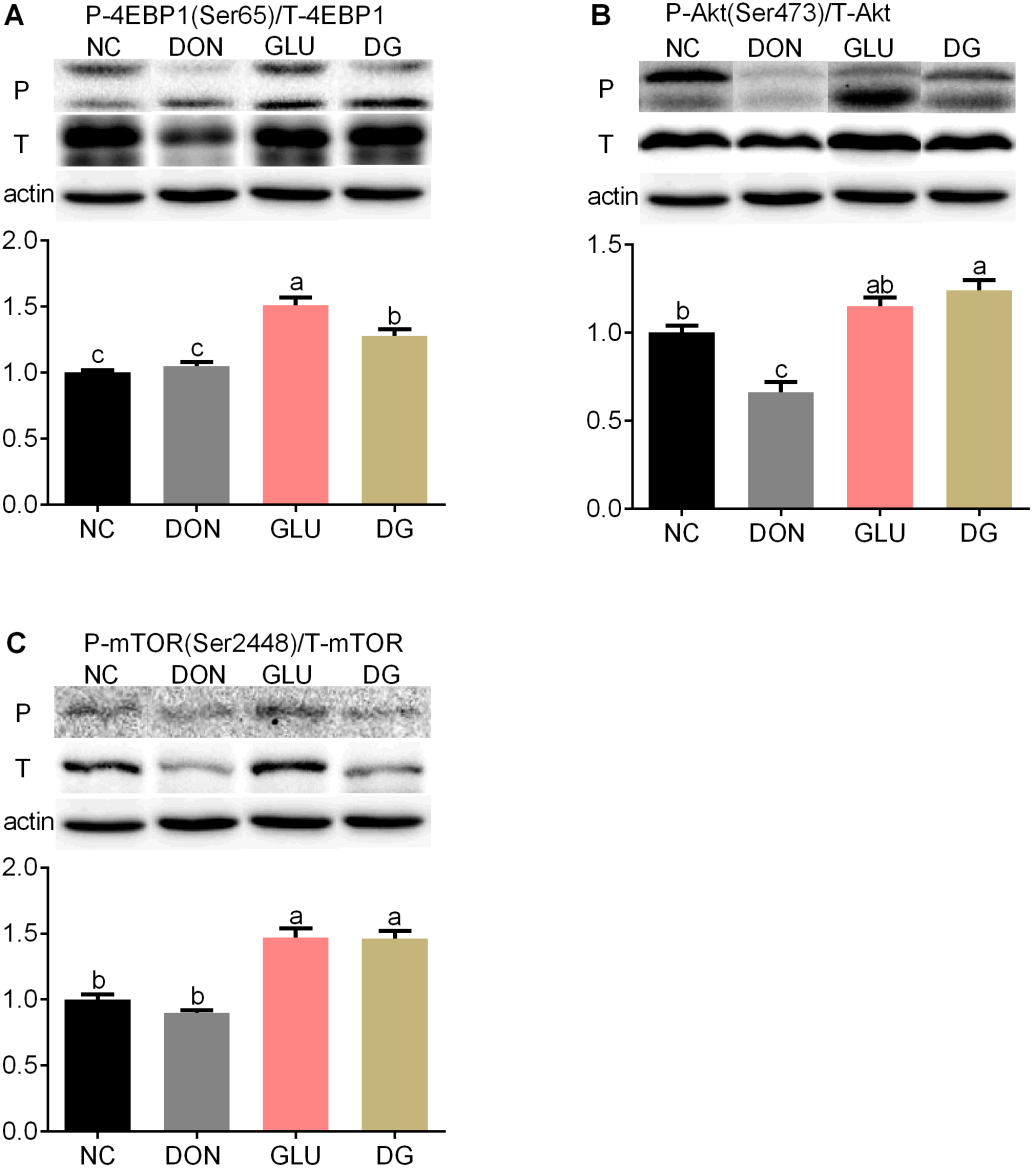
Activation of mTOR in ileum. (Up) Representative western blots of total 4EBP1(T-4EBP1), phosphorylated 4EBP1 (Ser65) (P-4EBP1), total Akt (T-Akt), phosphorylated Akt (Ser473) (P-Akt), total mTOR (T- mTOR), phosphorylated mTOR (Ser2448) (P- mTOR) in ileal of piglets fed the various dietary treatments. β-actin was the loading control. (Down) Quantification by image analysis of 4EBP1, Akt and mTOR phosphorylation. Dietary treatments were NC, an uncontaminated basal diet, DON, the basal contaminated with 4 mg/kg deoxynivalenol, GLU, uncontaminated basal diet with 2% (g/g) glutamic acid supplementation, and DG, deoxynivalenol-contaminated (4 mg/kg) basal diet with 2% (g/g) glutamic acid supplementation. n = 7 for treatments.

**Figure 8:**
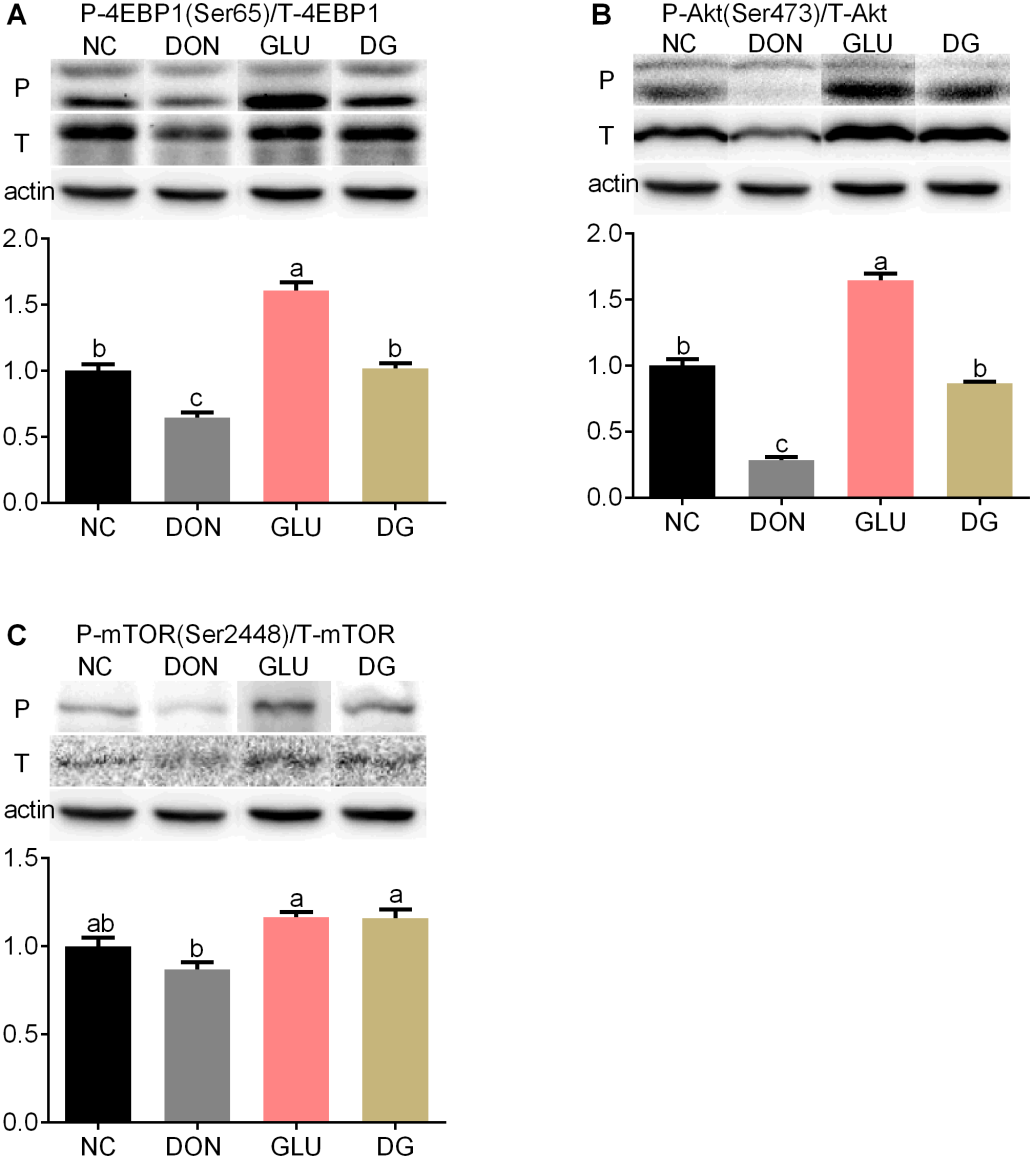
Activation of mTOR in jejunum. (Up) Representative western blots of total 4EBP1(T-4EBP1), phosphorylated 4EBP1 (Ser65) (P-4EBP1), total Akt (T-Akt), phosphorylated Akt (Ser473) (P-Akt), total mTOR (T- mTOR), phosphorylated mTOR (Ser2448) (P- mTOR) in jejunum of piglets fed the various dietary treatments. β-actin was the loading control. (Down) Quantification by image analysis of 4EBP1, Akt and mTOR phosphorylation. Dietary treatments were NC, an uncontaminated basal diet, DON, the basal contaminated with 4 mg/kg deoxynivalenol, GLU, uncontaminated basal diet with 2% (g/g) glutamic acid supplementation, and DG, deoxynivalenol-contaminated (4 mg/kg) basal diet with 2% (g/g) glutamic acid supplementation. n = 7 for treatments.

Although reports have suggested that many mycotoxins, including DON, may influence the MAPKs and/or PI3K signaling pathways [27], there is no evidence so far to show whether DON has an influence on the Akt/mammalian target of rapamycin (mTOR)/eukaryotic initiation factor 4e-binding protein 1(4EBP1) pathway. Previous studies reported that DON inhibits protein synthesis via binding to the 60S subunit of eukaryotic ribosomes [28] or activating the mitogen-activated protein (MAPK) signaling pathway [29], [30]. Therefore, in the present study we investigated the protein expression levels of 4EBP1, p-4EBP1, Akt, p-Akt, mTOR and p-mTOR in the ileum and jejunum so as to elucidate the detailed mechanisms by which DON induces cell death. As shown in Figure 7 and 8, feeding the DON diet reduced (P<0.05) phosphorylation levels and total protein abundance of Akt in the ileum (Figure 7) and jejunum (Figure 8), and phosphorylation levels of 4EBP1 in the jejunum (Figure 8), compared with feeding the NC diet. Glutamic acid supplementation increased (P<0.05) the protein phosphorylation of 4EBP1 by 21.3% in the ileum (Figure 7) and 57.4% in the jejunum (Figure 8), the protein phosphorylation of Akt by 86.8% (P<0.05) in the ileum (Figure 7) and 203.2% (P<0.05) in the jejunum (Figure 8), the phosphorylation of mTOR by 61.5% in the ileum (Figure 7) and 33.2% in the jejunum (Figure 8), compared with the DON diet.

## Discussion

CAT activity, T-AOC, H_2_O_2_, NO and MDA levels are widely accepted markers for oxidative stress [22], [31]–[34]. The present study showed that consuming DON-contaminated diets causes obvious oxidative stress to piglets from the change of analyzed indicators. Indeed, DON induced oxidative stress is widely observed in chickens [35], mice [36], pigs [13], rats [37], fish [38], and even cell lines isolated from human [39]. Intriguingly, our results demonstrated that supplementing glutamic acid to DON-contaminated piglet diet alleviates the oxidative stress caused by DON from the change of CAT, T-AOC, MDA and H_2_O_2_. It is well established that glutamate is involved in the oxidative response in body because L-glutamate is a precursor for glutathione, which is involved in the enterocyte redox state and in the detoxification process in enterocytes [40]. Further data about the serum GSH levels are needed to validate this explanation.

Reduced DAO activity in the intestine and kidney, and increased D-lactate level in serum are shown to correlate with the extent of histologic injury [41], [42]. Thus, in addition to microscopic lesions in intestine, the D-lactate concentration in serum, and DAO activity in serum and tissue were also detected to assess the effects of DON exposure on intestinal barrier function. The increased blood D-lactate levels, and reduced intestinal and kidney DAO activity suggest that the intestinal barrier integrity is severely compromised by DON intake. This reasoning also is demonstrated by the microscopic observation in the jejunum and ileum. The reasonable contributors come from the effect DON on wall morphology, tight junction, inflammation, oxidative stress, epithelial proliferation [35], [43], [44]. Interestingly, DON affects the intestinal epithelial barrier from both the apical (luminal) and basolateral (serosal) side [45]. Compellingly, supplementing glutamic acid to DON-contaminated diets promotes the intestinal recovery in piglets. In fact, increasing investigations in vivo and in vitro have demonstrated that glutamic acids exerts significant beneficial effects on intestinal barrier function [46], [47]. This intestinal barrier dysfunction might be associated with intestinal metabolism because the concentration of amino acid has a downward trend in DON group, while seven amino acids in DG group are increased, compared to DON group. The possible explanation is that glutamic acid, in addition to being used for protein synthesis within the intestinal mucosa, can also be used by enterocytes to produce other amino acids [48], [49].

The mTOR is a 289 kDa serine/threonine kinase, which plays a key role in regulating cell growth and proliferation [50]. Moreover, 4EBP1 is phosphorylated by mTOR to regulate translation initiation [51], whereas Akt contributes to mTOR activation [52]. Results showed that these proteins are significantly down-regulated after exposure to DON, indicating DON inhibits protein synthesis, consequently leading to decreased cell proliferation [53], [54]. Previous report showed several mycotoxins, particularly DON, causes the cell cycle arrest in the Gap2/Metaphase (G_2_/M) phase [55]–[57]. The G_2_/M arrest in intestinal epithelium cells caused by DON is associated with its inhibition on Akt/mTOR pathway because the inhibition of Akt/mTOR induces G_2_/M arrest in cells [58]. The levels of these proteins are up-regulated with dietary glutamic acid supplementation (DG group). This indicated that glutamic acid contributes to protein synthesis by regulating cell cycles. Indeed, previous studies have shown that glutamic acid appears to be involved in cellular signaling and growth regulation [15], [59], [60].

Taken together, our findings demonstrated that DON induces oxidative stress, and increases intestinal permeability, as well as inhibits protein synthesis and cell proliferation in weaned piglets. Glutamic acid supplementation decreases the oxidative stress and the intestinal permeability, and reverses Akt/mTOR/4EBP1 signaling caused by DON, indicating glutamic acid is a useful nutritional regulator for DON damage.
